# Neurodevelopmental outcome at two years of age and predictive value of General Movement Assessment in infants exposed to alcohol and/or drugs during pregnancy: a prospective cohort study

**DOI:** 10.1186/s12887-024-05046-w

**Published:** 2024-09-21

**Authors:** Toril Fjørtoft, Merethe Brandal, Lars Adde, Siril Osland, Hilde Rygh, Tordis Ustad, Kari Anne I. Evensen

**Affiliations:** 1 Clinic of Rehabilitation, St. Olavs Hospital, Trondheim University Hospital, Trondheim, Norway; 2 Department of Clinical and Molecular Medicine, Norwegian University of Science and Technology, Trondheim, Norway; 3 Children’s Clinic, St. Olavs Hospital, Trondheim University Hospital, Trondheim, Norway; 4 Department of Rehabilitation Science and Health Technology, Oslo Metropolitan University, Oslo, Norway

**Keywords:** Alcohol exposure, Drug exposure, Early infant behaviour, Neurodevelopment, Cognition, Language, Motor skills

## Abstract

**Background:**

Exposure to alcohol and/or other addictive drugs in pregnancy is a documented risk factor for neurological impairment. We aimed to assess neurodevelopmental outcome at two years of age in infants exposed to prenatal alcohol and/or other addictive drugs and to examine the predictive value of early motor assessment.

**Methods:**

This was a follow-up at two years of age in the prospective cohort study Children Exposed to Alcohol and/or Drugs in Intrauterine Life (CEADIL). The exposed group comprised 73 infants recruited from primary health care and included in a hospital follow-up programme at St. Olavs Hospital, Trondheim University Hospital, Norway. The control group comprised 93 healthy, unexposed infants recruited from the maternity ward at the same hospital. All children had been assessed by physiotherapists using the General Movement Assessment (GMA) at three months of age. Presence of fidgety movements, movement character and the Motor Optimality Score – Revised (MOS-R) were used. At two years of age, the children were assessed by trained examiners using the Bayley Scales of Infant and Toddler Development – Third Edition (BSID-III), Ages & Stages Questionnaires: Social-Emotional (ASQ:SE) and the Hollingshead Two-Factor Index of Social Position (SES).

**Results:**

The cognitive, language and motor composite scores of BSID-III were considerably lower in the exposed group than in the control group. Mean differences adjusted for age and parental SES ranged from − 13.3 (95% confidence interval, CI: -18.6 to -8.0) to -17.7 (95% CI: -23.3 to -12.2). Suboptimal fidgety movements and monotonous movement character had high sensitivity (0.94 to 0.74), but low specificity (0.10 to 0.32), while sensitivity and specificity of the MOS-R was around 50 and 60%, respectively.

**Conclusions:**

Neurodevelopmental outcome at two years of age was poorer in a group of children exposed to alcohol and/or drugs in pregnancy compared with a control group of healthy, unexposed children. Sensitivity of suboptimal fidgety movements and monotonous movement character at three months of age for later neurodevelopmental outcome was high to acceptable, but the MOS-R had limited sensitivity.

## Background

The use of addictive substances, especially alcohol, during pregnancy may lead to developmental disturbances of the foetus resulting in cognitive and behavioural problems and disabilities of the child [1–3]. Thus, substance use during pregnancy represents a major global child health issue [2]. Even if alcohol is the most widely used drug, alcohol abuse is often combined with abuse of opiates and/or other addictive substances or psychoactive drugs [4]. Use of cannabis in pregnancy has been discouraged because of the risk of adverse outcome for the child [5, 6].

Clinical outcome studies of drug abuse yield different results. In systematic reviews, adverse effects of benzodiazepines is not well documented [7], and no evidence of an association between prenatal cocaine exposure and developmental impairments has been found in children aged six years or younger [8]. However, a study including two-year-old children who were prenatally exposed to cocaine reported significantly poorer motor skills among these children compared with controls [9]. Another review reported statistically significant but subtle decrements in neurobehavioural, cognitive and language function up to three years in children exposed to cocaine use in pregnancy [10]. More recent studies have reported associations between prenatal cocaine exposure and cognitive and behavioural development also in adolescents [11, 12]. Similar effects have been described in adolescents exposed to amphetamine prenatally [13]. Prenatal exposure to methamphetamine was observed to have a negative but transient effect on fine motor performance in one-year-old children [14], while later studies are less conclusive [15]. In a study of six-year-old children exposed to amphetamine, heroin, benzodiazepines, cocaine and/or alcohol, 19% of the children had behavioural and concentration problems [16].

In a prospective cohort study, we assessed 108 infants exposed to alcohol and/or addictive drugs in pregnancy and 106 unexposed infants at three to four months of age using the Motor Optimality Score (MOS) of GMA [17] and the Alberta Infant Motor Scale (AIMS) [18]. We found an abnormal movement character in as many as two-thirds of the exposed infants and almost half had AIMS scores below the 10th percentile [19]. The effect of prenatal alcohol and/or drug exposure on motor repertoire, causing monotony and lack of movement variation could possibly be temporarily, or it could be more permanent. Longer term follow-up is therefore necessary.

Early detection of neurodevelopmental impairments is a prerequisite for early and focused intervention [20], and a tool for prediction of outcome in children exposed to addictive substances in pregnancy is essential. In preterm and high-risk infants, a detailed analysis of infants’ movement patterns by using the Prechtl GMA can predict cerebral palsy [20, 21] and later cognitive dysfunction [22, 23]. However, few have examined the predictive value of early motor assessments in drug-exposed children. A study of children exposed to opiates in pregnancy showed that abnormal spontaneous movements in infancy pose a high risk for later neurological difficulties [24]. Another study examining children born to mothers who were treated with antiepileptic drugs in pregnancy found that early assessment with GMA correlated with psychomotor development at 30 months [25].

The primary aim of the present follow-up study was to determine neurodevelopmental outcome in terms of cognitive, language and/or motor function at two years of age in the group of exposed children. The secondary aim was to examine whether motor behaviour at three months could predict cognitive, language and/or motor function at two years of age.

## Methods

### Study design

The present study was a follow-up at two years of age in the prospective cohort study Children Exposed to Alcohol and/or Drugs in Intrauterine Life (CEADIL). The study included a group of children exposed to prenatal maternal abuse of alcohol and/or addictive drugs and a control group of healthy, unexposed children. Details of the cohort have been described in a previous publication of the effect of alcohol and drug use on motor behaviour and general movements in infancy [19]. The exposed children were born to mothers who had been identified by the primary health care and social services as substance abusers during pregnancy. They were recruited at three months of age when they were included in a follow up program of children at risk for impaired development at St. Olavs Hospital, Trondheim University Hospital, Norway, between 6th of December 2014 and 5th of May 2019. The control group was recruited consecutively from the Maternity ward at St. Olavs Hospital, Trondheim University Hospital, Norway between February and December 2018. The two-year follow-up was carried out between September 2017 and August 2021.

### Participants

#### Exposed group

**Fig. 1:**
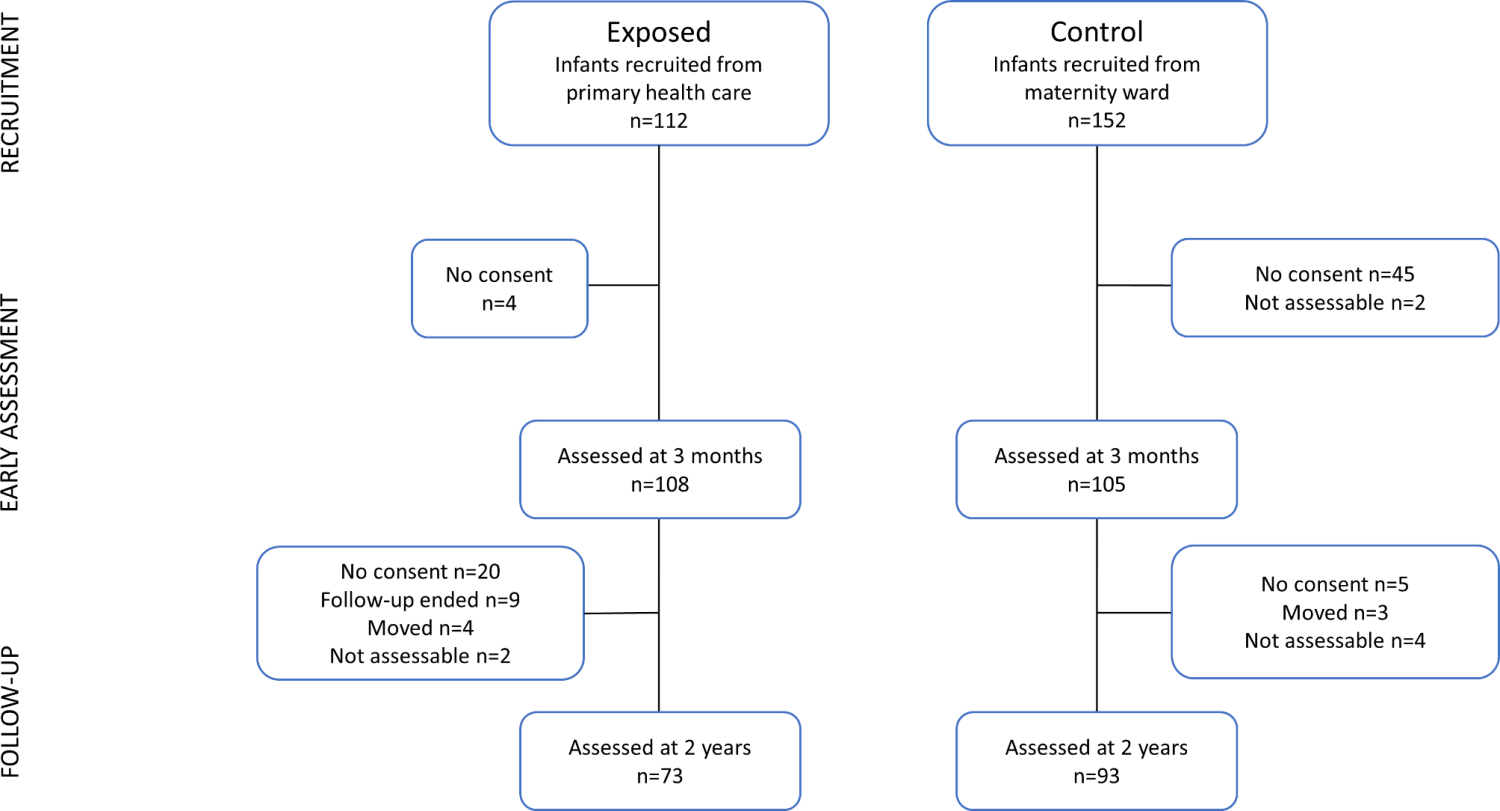
Flow of participants

Flow of participants is shown in Fig. 1. A total of 112 children were invited to participate at three months of age. Parents of 108 children gave written consent to participate on behalf of their child. At two years of age, parents of 20 children did not consent to participation, follow-up by the team had ended for nine children, four children had moved and were unable to meet for assessment and two children did not cooperate during assessment. Thus, 73 children in the exposed group were assessed at two years of age.

#### Control group

The control group included 105 children recruited in infancy. At two years of age, parents of five children did not consent to participation, three children had moved and were unable to meet for assessment and four children did not cooperate during assessment. Thus, 93 children in the control group were assessed at two years of age.

### Non-participants

There were no significant differences in gestational age, birth weight, head circumference and length at birth or sex distribution between participants and non-participants in either group.

### Background information

Data on gestational age, birth weight, head circumference and length at birth were retrieved from hospital records. Information regarding maternal abuse during pregnancy was self-reported by the mothers [19]. Socioeconomic status (SES) of either biological or foster parents at follow-up was calculated for both groups using the Hollingshead Two-Factor Index of Social Position, which is based on education and occupation of one parent or the mean index of both [26].

### Video recordings and Motor Optimality Score for 3-to 5-Month-Old Infants – Revised

The infants’ spontaneous movements were video recorded at a mean age of 53.1 (standard deviation [SD] 1.4) weeks postmenstrual age in the exposed group and 53.6 (SD 1.3) weeks postmenstrual age in the control group. They were assessed blindly by four GMA-certified, experienced paediatric physiotherapists in compliance with the procedure described by Einspieler et al. [27]. The videos of both groups were re-identified, mixed, and assessed along with videos of other infants recorded for routine clinical purposes. The observers evaluated the footage separately, and the project coordinator administered videos to a third observer if the first two observers disagreed. The quality of the fidgety movements was classified as optimal (F++) or suboptimal if the fidgety movements were intermittent (F+), sporadic (F+/-), abnormal (FA) or absent (F-) [28]. In this study, none of the infants had absent fidgety movements (F-). Optimal fidgety movements (F++) were seen in 17 (16%) infants in the exposed group and 43 (41%) infants in the control group. Motor Optimality Score (MOS) was calculated for each infant. As a revised version of the MOS was recently published [27], data were reassessed according to the guidelines of this version, where the third subcategory (age-adequate movement repertoire) is scored according to the infant’s postmenstrual age. The Motor Optimality Score – Revised (MOS-R) has a cut-off score of ≤ 24 points [27].

### Bayley Scales of Infant and Toddler Development – Third Edition

The children were assessed by trained examiners using the Bayley Scales of Infant and Toddler Development – Third Edition (BSID-III) [29]. The BSID-III is one of the most widely used standardised scales to measure cognitive and motor function in infants and toddlers. Age-adjusted composite scores were calculated for cognitive, language and motor domains. The BSID-III language and motor composite scores are derived from sums of the scaled subtest scores, whereas the BSID-III cognitive composite score is derived from a single scaled score. Composite scores range from 40 to 160, with a mean of 100 and a SD of 15. Poor neurodevelopmental outcome was defined as a score below 1 SD of the mean in the control group.

### Ages & Stages Questionnaires: Social-Emotional (ASQ:SE)

ASQ:SE is a set of questionnaires about children’s social-emotional development from 6 to 60 months which can be self-administered by parents/caregivers [30]. It is used as a screening tool to identify infants and young children whose social and emotional development require further evaluation, and to determine if these children should be referred to intervention services. Eight questionnaires are available for different age groups and in this study, most children were assessed by the 24- or 30-months questionnaire, and five children were assessed by the 36-months questionnaire. The questionnaires screen for self-regulation, compliance, communication, adaptive behaviours, autonomy, affect, and interaction with people. The biological or foster parents filled out the questionnaire and the paediatric physiotherapist was available to answer questions while they completed it. Higher scores indicate more concerns. We used the cut-offs specified on the scoring sheets, corresponding to a cut-off above 50 points on the 24-months questionnaire, 57 points on the 30-months questionnaire and 59 points on the 36-months questionnaire [30].

### Statistical analyses

Data were analysed with SPSS Statistics, version 29.0 (IBM SPSS Statistics, Chicago, IL, USA). Group differences were analysed using the Chi-square test and differences in non-parametric data were analysed by using the Mann-Whitney *U* test. Sensitivity, specificity and predictive values of suboptimal fidgety movements, monotonous movement character and MOS-R ≤ 24 points for later neurodevelopmental outcomes were calculated by cross tables. Ninety-five percent confidence intervals (CI) were calculated using the Wilson method, as recommended by Altman [31].

## Results

### Clinical characteristics

Clinical characteristics are shown in Table 1. There were no differences between the exposed group and the control group in weight, head circumference or height at birth. Mean age at follow-up was 2.3 years in both groups. Parental SES was lower in the exposed group. In the exposed group, 17 children were residents in foster homes and mothers of 45 children had reported alcohol, benzodiazepine, cannabis, or amphetamine abuse.

**Table 1:** Clinical characteristics of the exposed group and the control group at birth and at follow-up

	Exposed			Control		
	n	Mean	(SD)	n	Mean	(SD)
Gestational age (weeks)	72	38.8	(1.8)	92	39.8	(0.8)
Birth weight (g)	72	3321	(626)	93	3570	(457)
Head circumference (cm)	68	34.6	(1.7)	92	35.5	(1.6)
Length (cm)	69	48.6	(2.6)	89	50.2	(2.0)
Postmenstrual age at early assessment (weeks)	73	53.1	(1.4)	93	53.6	(1.3)
Age at follow-up assessment (years)	73	2.3	(0.4)	93	2.3	(0.2)
Parental SES at follow-up	72	2.1	(1.4)	92	4.2	(0.9)
		n	(%)		n	(%)
Boys	73	41	(56)	93	38	(41)
Foster homes	73	17	(23)	93	0	(0)
Alcohol, benzodiazepine, cannabis, or amphetamine abuse	73	45	(62)			
Prescription drugs for treatment and/or addiction	73	19	(26)			
Opioid replacement therapy	73	5	(7)			
Identified as active abusers by primary health care	73	4	(5)			

SD = Standard deviation; SES = Socioeconomic status

### Neurodevelopmental outcome at two years

Adjusted for sex and parental SES, the exposed group had lower BSID-III subscores as well as cognitive, language and motor composite scores compared with the control group (Table 2). They also had lower ASQ:SE scores assessed by the 24-months, but not by the 30-months, questionnaire. In total, ten (14.1%) children in the exposed group had a score above cut-off compared with only one child in the control group (Table 3).

**Table 2:** Bayley Scales of Infant and Toddler Development – Third Edition (BSID-III) and Ages and Stages Questionnaires: Social-Emotional (ASQ:SE) scores at follow-up

Total and subtest scores	Exposed			Control			Mean difference adjusted for Ages & Stages (95% CI)		*p*-value	Mean difference adjusted for sex and SES (95% CI)		*p*-value
	*n*	Mean	(SD)	*n*	Mean	(SD)						
BSID-III Cognitive Scaled Score	73	9.4	(2.1)	93	12.8	(2.9)	-3.4	(-4.2 to -2.6)	< 0.001	-3.6	(-4.7 to -2.4)	< 0.001
BSID-III Receptive Language Scaled Score	73	8.2	(2.1)	93	11.2	(2.6)	-2.9	(-3.7 to -2.2)	< 0.001	-3.1	(-4.1 to -2.1)	< 0.001
BSID-III Expressive Language Scaled Score	72	8.8	(2.3)	93	11.2	(2.4)	-2.3	(-3.0 to -1.6)	< 0.001	-2.9	(-3.9 to -1.9)	< 0.001
BSID-III Sum Language Scaled Score	72	16.9	(4.2)	93	22.4	(4.6)	-5.2	(-6.6 to -3.8)	< 0.001	-6.0	(-7.9 to -4.2)	< 0.001
BSID-III Fine Motor Scaled Score	73	10.3	(2.8)	93	12.7	(2.8)	-2.2	(-3.0 to -1.3)	< 0.001	-2.7	(-3.9 to -1.6)	< 0.001
BSID-III Gross Motor Scaled Score	72	7.3	(1.9)	92	9.0	(2.3)	-1.7	(-2.4 to -1.0)	< 0.001	-1.9	(-2.9 to -1.0)	< 0.001
BSID-III Sum Motor Scaled Score	72	17.8	(3.9)	92	21.7	(4.3)	-3.7	(-5.0 to -2.4)	< 0.001	-4.4	(-6.2 to -2.6)	< 0.001
BSID-III Cognitive Composite Score	73	96.9	(10.5)	93	114.2	(14.7)	-17.1	(-21.2 to 13.0)	< 0.001	-17.7	(-23.3 to -12.2)	< 0.001
BSID-III Language Composite Score	72	91.1	(12.1)	93	107.1	(13.6)	-15.4	(-19.4 to -11.3)	< 0.001	-17.6	(-23.0 to -12.2)	< 0.001
BSID-III Motor Composite Score	72	93.6	(11.9)	92	105.4	(12.9)	-11.3	(-15.2 to -7.4)	< 0.001	-13.3	(-18.6 to -8.0)	< 0.001
ASQ:SE 24 months Total Score	40	25.4	(21.5)	31	12.9	(8.5)	9.9	(1.6 to 18.3)	0.020	13.7	(3.7 to 23.7)	0.008
ASQ:SE 30 months Total Score	27	36.5	(33.5)	61	28.4	(15.9)	8.1	(-2.4 to 18.6)	0.127	10.9	(-4.4 to 26.3)	0.161

ASQ:SE = Ages & Stages Questionnaires: Social-Emotional; BSID-III = Bailey Scales of Infant and Toddler Development – Third Edition; SD = Standard deviation; SES = Socioeconomic status

Odds ratio for scoring below 1 SD on the cognitive, language and motor composite scores of the BSID-III and above cut-off on the ASQ:SE was higher in the exposed group, adjusted for sex (Table 3). The odds ratios were even higher when we additionally adjusted for parental SES. Of the 17 exposed children living in foster homes, all had a BSID-III score below cut-off and/or an ASQ:SE score above cut-off.

**Table 3:** Proportion of children and odds ratio for scoring below cut-off on the Bayley scales of Infant and Toddler Development – Third Edition (BSID-III) and above cut-off on the Ages & Stages Questionnaire: Social-Emotional (ASQ:SE)

Total and subtest scores	Exposed (*n* = 73)		Control (*n* = 93)		OR adjusted for sex (95% CI)		*p*-value	OR adjusted for sex and SES (95% CI)		*p*-value
	*n*	(%)	*n*	(%)						
BSID-III Cognitive < 1 SD	35	(47.9)	13	(14.0)	5.6	(2.6 to 11.8)	< 0.001	8.1	(3.0 to 22.1)	< 0.001
BSID-III Language < 1 SD^a^	37	(51.4)	15	(16.1)	5.3	(2.6 to 11.0)	< 0.001	11.6	(4.0 to 33.6)	< 0.001
BSID-III Motor < 1 SD^b^	31	(43.1)	16	(17.4)	3.6	(1.7 to 7.3)	< 0.001	5.2	(2.0 to 14.0)	< 0.001
ASQ:SE > cut-off^c^	10	(14.1)	1	(1.1)	13.4	(1.7 to 108.0)	0.015	31.2	(3.1 to 316.2)	0.004

ASQ:SE = Ages & Stages Questionnaires: Social-Emotional; BSID-III = Bailey Scales of Infant and Toddler Development – Third Edition; CI = Confidence interval; OR = Odds ratio; SD = Standard deviation; SES = Socioeconomic status

^a^Data on BSID-III Language Composite Score missing for one participant in the exposed group

^b^Data on BSID-III Motor Composite Score missing for one participant in the exposed group and one participant in the control group

^c^Data on ASQ:SE missing for two participants in the exposed group and one participant in the control group

### Predictive value of early motor assessment for neurodevelopmental outcome at two years

Table 4 presents sensitivity, specificity, and predictive values of suboptimal fidgety movements (F+, F+/-, FA) at three months of age for neurodevelopmental outcome expressed as BSID-III scores below 1 SD and ASQ:SE score above cut-off at two years of age in the exposed group. Sensitivity of suboptimal fidgety movements was 0.94 for the cognitive and motor composite scores, 0.92 for the language composite score and 0.90 for ASQ:SE, while specificity was very low (0.10–0.13). PPV ranged from 0.14 to 0.52 and NPV ranged from 0.57 to 0.86.

**Table 4:** Predictive values of suboptimal fidgety movements (F+, F+/-, FA) for poor neurodevelopmental outcome in exposed children

	*n*	Sensitivity	(95% CI)	Specificity	(95% CI)	PPV	(95% CI)	NPV	(95% CI)
BSID-III Cognitive < 1 SD	73	0.94	(0.81 to 0.98)	0.13	(0.06 to 0.27)	0.50	(0.38 to 0.62)	0.71	(0.36 to 0.92)
BSID-III Language < 1 SD	72	0.92	(0.79 to 0.97)	0.11	(0.05 to 0.26)	0.52	(0.40 to 0.64)	0.57	(0.25 to 0.84)
BSID-III Motor < 1 SD	72	0.94	(0.79 to 0.98)	0.12	(0.05 to 0.26)	0.45	(0.33 to 0.57)	0.71	(0.36 to 0.92)
ASQ:SE > cut-off	71	0.90	(0.60 to 0.98)	0.10	(0.05 to 0.20)	0.14	(0.08 to 0.25)	0.86	(0.49 to 0.97)

ASQ:SE = Ages & Stages Questionnaires: Social-Emotional; BSID-III = Bailey Scales of Infant and Toddler Development – Third Edition; CI = Confidence interval; NPV = Negative predictive value; PPV = Positive predictive value; SD = Standard deviation

Tables 5 and 6 presents sensitivity, specificity, and predictive values of monotonous movement character and MOS-R ≤ 24 and at three months of age for neurodevelopmental outcome at two years. The sensitivity of monotonous movement character ranged from 0.74 to 0.80, while specificity was lower (0.29 to 0.32). Sensitivity of MOS-R ≤ 24 points ranged from 0.40 to 0.54 and specificity from 0.52 to 0.58. PPV and NPV of both monotonous movement character and MOS-R ≤ 24 points were around 50 to 60%, except for ASQ:SE where PPV was lower and NPV higher.

**Table 5:** Predictive values of monotonous movement character for poor neurodevelopmental outcome in exposed children

	*n*	Sensitivity	(95% CI)	Specificity	(95% CI)	PPV	(95% CI)	NPV	(95% CI)
BSID-III Cognitive < 1 SD	73	0.77	(0.61 to 0.88)	0.32	(0.19 to 0.47)	0.51	(0.38 to 0.64)	0.60	(0.39 to 0.78)
BSID-III Language < 1 SD	72	0.76	(0.60 to 0.87)	0.31	(0.19 to 0.48)	0.54	(0.41 to 0.67)	0.55	(0.34 to 0.74)
BSID-III Motor < 1 SD	72	0.74	(0.57 to 0.86)	0.29	(0.18 to 0.44)	0.44	(0.32 to 0.58)	0.60	(0.39 to 0.78)
ASQ:SE > cut-off	71	0.80	(0.49 to 0.94)	0.30	(0.20 to 0.42)	0.16	(0.08 to 0.28)	0.90	(0.70 to 0.97)

ASQ:SE = Ages & Stages Questionnaires: Social-Emotional; BSID-III = Bailey Scales of Infant and Toddler Development – Third Edition; CI = Confidence interval; NPV = Negative predictive value; PPV = Positive predictive value; SD = Standard deviation

**Table 6:** Predictive values of the Motor Optimality Score – Revised ≤ 24 points for poor neurodevelopmental outcome in exposed children

	*n*	Sensitivity	(95% CI)	Specificity	(95% CI)	PPV	(95% CI)	NPV	(95% CI)
BSID-III Cognitive < 1 SD	73	0.54	(0.38 to 0.70)	0.58	(0.42 to 0.72)	0.54	(0.38 to 0.70)	0.58	(0.42 to 0.72)
BSID-III Language < 1 SD	72	0.54	(0.38 to 0.69)	0.57	(0.41 to 0.72)	0.57	(0.41 to 0.72)	0.54	(0.38 to 0.69)
BSID-III Motor < 1 SD	72	0.52	(0.35 to 0.68)	0.56	(0.41 to 0.70)	0.47	(0.31 to 0.63)	0.61	(0.45 to 0.74)
ASQ:SE > cut-off	71	0.40	(0.17 to 0.69)	0.52	(0.40 to 0.64)	0.12	(0.05 to 0.27)	0.84	(0.70 to 0.93)

ASQ:SE = Ages & Stages Questionnaires: Social-Emotional; BSID-III = Bailey Scales of Infant Development – Third Edition; CI = Confidence interval; NPV = Negative predictive value; PPV = Positive predictive value; SD = Standard deviation

## Discussion

In this follow-up of a prospective cohort study, we found that neurodevelopment in terms of cognitive, language and motor skills as well as social and emotional development at two years of age was poorer in a group of children exposed to alcohol and/or drugs compared with healthy control children. The presence of suboptimal fidgety movements at three months of age seemed to identify almost all exposed children with poor neurodevelopmental outcome at follow-up. Monotonous movement character identified up to 80% of the children, while a MOS-R ≤ 24 points only identified about half of the children.

The strengths and limitations regarding the exposed group have been discussed in detail in a previous paper [19]. Primary health care applied strict criteria for identifying pregnant women at risk, and it is therefore reasonable to believe that all infants in the exposed group had been exposed to alcohol and/or other addictive substances in utero. In the present follow-up study, 35 of 108 children were not assessed, corresponding to a dropout rate of 32%. Considering the social complexity of the exposed group, less dropout is hard to achieve. Moreover, there were no differences in background characteristics between participants and non-participants.

A limitation of this study is the heterogeneity of the exposed group with respect to alcohol and/or drug exposure. Even if the significant difference in neurodevelopment at two years of age between the exposed and the control group is most likely due to an intrauterine effect of substance abuse, it is impossible to differentiate between results according to the type of abuse.

In the present study, results were adjusted for parental SES as SES is associated with a wide array of health and cognitive outcomes in children [32]. Differences in child development could also been explained by parental practises and ability [33]. However, information of such variables could not be obtained in this study. Because the control group was recruited from the same maternity ward and local population as the exposed group, differences related to ethnicity and cultural variation are not likely.

While the assessments at three months were performed blinded to group status [19], this was not possible at follow-up. Children in the exposed group was assessed as part of a follow-up program for children at risk for impaired neurodevelopment, and consequently examiners were not blinded. This was done to avoid dropouts as parents were more likely to participate when the research project was integrated in their regular paediatric follow-up program. The control group was assessed separately.

Most children in our exposed group were born to mothers reporting a combination of alcohol and drug abuse. The poorer BSID-III motor scores found in our study are in line with a study reporting significantly poorer fine and gross motor skills in 99 two-year-old children who were prenatally exposed to cocaine compared with 101 controls, although they were assessed with the Peabody Developmental Motor Scales [10]. The findings of poorer BSID-III cognitive and language scores are supported by a meta-analysis of 2236 participants from six prospective longitudinal cohort studies in the United States reporting effects of prenatal alcohol exposure on cognitive and behavioural development in school age, adolescence and young adulthood [3] and a review reporting impaired neurobehavioural, cognitive and language function up to three years of age in children exposed to cocaine use in pregnancy [10].

Our findings that children with poor neurodevelopmental outcome were identified by suboptimal fidgety movements are consistent with a study from 2012 on 77 children exposed to opiate abuse and HIV in pregnancy [24]. In this study, the authors concluded that abnormal general movements in infancy could be utilised for early identification of the infants at risk for adverse neurodevelopmental outcome at two years. Also, despite having a smaller sample of 11 children born to mothers who were treated with antiepileptic drugs in pregnancy, Parisi et al. [25] found that abnormal general movements assessed at several timepoints in infancy correlated with poorer psychomotor development at 30 months.

The differences were still significant after adjusting for both sex and parental SES. Interestingly, all infants raised by foster parents had poor neurodevelopmental outcome. Even if these numbers are small, it could indicate that optimal care given by care givers selected based on good references and good parenting ability is not enough for reversing non-optimal neurodevelopment. It is therefore most likely that the differences observed have a biological explanation. Another explanation might be that the infants that had been most heavily exposed in utero also are the ones with biological parents least capable of offering optimal care and are therefore selected for foster homes.

In this study, suboptimal fidgety movements at three months of age seemed to identify almost all exposed children with poor neurodevelopmental outcome. As specificity was low, suboptimal fidgety movements also identified a large group of children with normal neurodevelopment (false positives). It is therefore questionable if quality of fidgety movements alone is useful for selecting children for early and focused intervention. Monotonous movement character identified up to 80%, but MOS-R identified only about half of the exposed children with poor neurodevelopmental outcome. However, specificity was higher indicating a lower number of false positives. More studies are needed to explore how useful the MOS-R is as a screening tool for this group of children.

## Conclusions

Neurodevelopment at two years of age in a group of children exposed to alcohol and/or drugs in pregnancy was considerably impaired compared with a control group, when assessed with the BSID-III and ASQ:SE. Sensitivity of suboptimal fidgety movements and monotonous movement character at three months for neurodevelopmental outcome at two years of age was high to acceptable, but the MOS-R had limited sensitivity. Further research is necessary for the best prediction of outcome.

## Data Availability

The datasets generated and/or analysed during the current study are not publicly available because permission has not been applied for from neither the participants nor the Ethical Committee but aggregated data may be available from the corresponding author on reasonable request.

## Abbreviations

ADHD: Attention Deficit Hyperactivity Disorder
AIMS: Alberta Infant Motor Scales
ASQ:SE: Ages & Stages Questionnaires: Social-Emotional
BSID-III: Bayley Scales of Infant and Toddler Development – Third Edition
CI: Confidence interval
GMA: General Movement Assessment
MOS: Motor Optimality Score
MOS-R: Motor Optimality Score – Revised
NPV: Negative predictive value
PPV: Positive predictive value
SD: Standard deviation
SES: Socioeconomic status
